# Validity of the New International Diabetes Federation-Diabetes and Ramadan (IDF-DAR) Risk Stratification Score and Fasting Experience of Saudi Patients With Diabetes During Ramadan: Insights From a Cross-Sectional Study

**DOI:** 10.7759/cureus.79351

**Published:** 2025-02-20

**Authors:** Mussa Almalki, Aseel A AlSaeed, Areej A AlNomi, Maram AlSufyani, Khalid Albedaiwi, Fahad Alshahrani, Ohoud AlMohareb, Naji Aljohani, Awad Alshahrani

**Affiliations:** 1 Obesity, Endocrine, and Metabolism Center, King Fahad Medical City, Riyadh, SAU; 2 College of Medicine, Alfaisal University, Riyadh, SAU; 3 Department of Primary Health Care, Ministry of National Guard - Health Affairs, Taif, SAU; 4 Department of Medicine, Ministry of National Guard - Health Affairs, Riyadh, SAU; 5 Department of Family and Community Medicine, King Abdullah International Medical Research Center, Riyadh, SAU; 6 Department of Family Medicine, Ministry of National Guard - Health Affairs, Riyadh, SAU; 7 College of Medicine, King Saud bin Abdulaziz University for Health Sciences, Riyadh, SAU

**Keywords:** diabetes, idf-dar risk stratification, ramadan, saudi arabia, validity

## Abstract

Background

The International Diabetes Federation-Diabetes and Ramadan (IDF-DAR) risk stratification tool is designed to predict adverse outcomes in patients with diabetes who fast during Ramadan.

Objectives

This study evaluates the accuracy of the IDF-DAR tool in predicting adverse outcomes among Saudi patients with diabetes intending to fast during Ramadan while comparing outcomes across different risk categories.

Methods

This prospective observational study was conducted at the Obesity, Endocrine, and Metabolism Center, King Fahad Medical City, and diabetes clinics at King Abdulaziz Medical City in Riyadh, Saudi Arabia. Data were collected through questionnaires on diabetes characteristics, complications, comorbidities, and factors influencing fasting. Ordinal regression analysis was performed to identify predictors of risk levels.

Results

The cohort consisted of 303 patients, including 163 females (53.5%) with a mean age of 50.49 ± 17.91 years. Type 2 diabetes mellitus was diagnosed in 217 participants (71.6%), and 231 patients (76.2%) had diabetes for over 10 years. Risk stratification categorized 39 patients (12.9%) as low risk, 71 (23.4%) as moderate risk, and 193 (63.7%) as high risk. Self-monitoring of blood glucose adherence was reported in 174 patients (57.4%), with the highest adherence in the high-risk group (126 or 65.3%) and the lowest in the low-risk group (18 or 46.2%). Hyperglycemia (>16.6 mmol/L) was observed in 141 participants (47%): 13 (33.3%) low risk, 26 (36.6%) moderate risk, and 103 (53.4%) high risk. Hypoglycemia was the most common reason for breaking the fast, notably among 23 patients (71.9%) fasting for more than 15 days. A significant majority of participants aimed to fast for the full 30 days, with the highest completion rate among moderate-risk individuals at 58 (81.7%). In contrast, 27 individuals (14.92%) in the high-risk group did not fast at all.

Conclusions

The IDF-DAR tool effectively stratifies fasting risk in diabetic patients but may not accurately reflect the risk for some high-risk individuals. Further validation is necessary to enhance its predictive accuracy.

## Introduction

Diabetes mellitus (DM) is a globally prevalent metabolic disorder with a significant burden in Saudi Arabia. A systematic review by Alwadeai and Alhammad reported a 28% prevalence of type 2 DM (T2DM) among Saudi adults [1]. The global diabetes burden is rising, with the International Diabetes Federation (IDF) projecting 783 million cases by 2045 [2,3]. During Ramadan, Muslims fast from dawn to sunset, abstaining from food and drink, with two main meals: Saher (pre-dawn) and Iftar (sunset) [4]. While exemptions exist for chronic illnesses, many individuals with diabetes choose to fast despite medical advice, increasing their risk of hypoglycemia, hyperglycemia, dehydration, and diabetic ketoacidosis (DKA) [5-8].

In 2021, the IDF-Diabetes and Ramadan (IDF-DAR) Alliance introduced a risk categorization tool to assess fasting risks in diabetic individuals [9]. The reliability and validity of this tool have been established through multiple epidemiological studies [10,11]. Recently, it has been validated in research conducted in the UAE and Bangladesh, showcasing its capacity to predict both fasting safety and the likelihood of hypo- or hyperglycemia [12,13]. However, these studies noted that many high-risk patients fasted successfully, suggesting a potential overestimation of risk. A study at the Diabetes Center of King Fahad Hospital, Saudi Arabia affirmed the tool’s validity but also indicated a possible overestimation of fasting risks [14]. Despite these findings, limited data exist on the tool’s predictive accuracy for adverse outcomes in Saudi Arabia, particularly in high-risk patients. This study aims to validate the IDF-DAR risk stratification tool in predicting adverse outcomes among Saudi diabetic patients fasting during Ramadan at two tertiary care centers in Riyadh and to compare outcomes across risk categories.

## Materials and methods

Study design

We conducted a prospective observational study at two centers: the Obesity, Endocrine, and Metabolism Center at King Fahad Medical City and the diabetes clinics at King Abdulaziz Medical City in Riyadh, Saudi Arabia.

Eligibility criteria and study subjects

Eligible participants were adults (both male and female) diagnosed with type 1 DM (T1DM) or T2DM who had undergone a risk assessment starting four weeks prior to Ramadan 2024. Participants needed to have attended diabetes clinics and provided consent to participate. We excluded patients with severe psychiatric illnesses, those who were critically ill and unable to fast or monitor their blood glucose, and pregnant women at the time of screening.

Data collection

Participants meeting the eligibility criteria were evaluated using an investigator-administered questionnaire that assessed diabetes characteristics, related complications, comorbidities, and factors influencing fasting. Participants were required to maintain a daily log throughout Ramadan, recording whether they completed the fast, their reasons for breaking the fast, and the timing of such breaks. Instances of hypoglycemia or hyperglycemia were to be recorded with corresponding blood glucose levels. Sociodemographic data, including age and sex, were also collected. Using the IDF-DAR Risk Stratification Score (as detailed in Appendix A), participants were categorized into three risk groups: low risk (score 0-3), moderate risk (score 3.5-6), and high risk (score >6). These stratifications were compared with observed outcomes, such as fasting-related hypoglycemia and other adverse events, using descriptive statistics and comparative analyses. Additionally, patient-reported experiences and behaviors during fasting were analyzed to provide practical insights into the tool’s applicability to the local population. Fasting guidelines based on these risk levels advised low-risk individuals to fast, while moderate-risk and high-risk individuals were advised against it. Participants’ intentions to fast or not were assessed according to their risk categories and recommendations. All participants received Ramadan-focused diabetes education, with personalized adjustments to diet, physical activity, and glucose-lowering medications. Follow-up was conducted within one month post-Ramadan through in-person or phone interviews. Post-Ramadan data included the number of fasting days, adherence to dietary and lifestyle recommendations, medication adjustments, self-monitoring of blood glucose (SMBG), and occurrences of hypoglycemia (<3.9 mmol/L) and hyperglycemia (>16.6 mmol/L). Plasma glucose levels after fasting and two hours post-meal were measured if available. SMBG was assessed via participant-reported finger-prick tests. Continuous glucose monitoring system (CGMS) profiles were not used for comparison.

Statistical analysis

Descriptive statistics summarized continuous variables as means with SDs and categorical variables as frequencies with proportions, analyzing baseline characteristics of individuals who completed follow-up. Graphs illustrated fasting days achieved based on pre-Ramadan risk categories and hypoglycemia incidence, stratified by pre-Ramadan IDF-DAR scores. Statistical analyses used chi-square or Fisher’s exact tests (p < 0.05) for small sample-size cells. Ordinal regression identified predictors for Ramadan risk categories (low, moderate, and high), modeling the odds of being in a higher-risk category. The model’s goodness of fit and explanatory power were evaluated using Pearson and deviance chi-square statistics and pseudo-R-square measures. Analyses were conducted using IBM SPSS Statistics for Windows, Version 26.0 (Released 2019; IBM Corp., Armonk, NY, USA).

## Results

A total of 303 patients were enrolled in the study, including 163 females (53.5%) with a mean age of 50.49 ± 17.91 years. The majority, 217 patients (71.6%), had T2DM, and 231 patients (76.2%) had diabetes for more than 10 years. Glycemic control was categorized based on glycated hemoglobin (HbA1c) levels: 70 participants (23.1%) had good control (HbA1c <7.5%), 154 participants (50.8%) had moderate control (HbA1c 7.5-9.0%), and 79 participants (26.1%) had uncontrolled diabetes (HbA1c >9.0%). Additionally, only 15 participants (5%) had an estimated glomerular filtration rate (eGFR) <45 mL/min, while 267 patients (88.1%) had an eGFR >60 mL/min. The baseline characteristics of individuals who completed the follow-up are detailed in Table 1.

**Table 1:** Baseline characteristics of the study population One-way ANOVA was used to compare the age across three groups; for all other values, test statistics refer to chi-square (cell count for expected frequency >5) or Fisher’s exact (expected frequency in each cell is <5) test whenever applicable. eGFR, estimated glomerular filtration rate; LADA, latent autoimmune diabetes in adults; MODY, maturity-onset diabetes of the young; T1DM, type 1 diabetes mellitus; T2DM, type 2 diabetes mellitus

Variable	Subgroups	Total (n = 303)	Low risk (n = 39)	Moderate risk (n = 71)	High risk (n = 193)	Test statistic	p-value
Age	Range (14-82 years)	50.49 ± 17.91	55.87 ± 13.19	55.41 ± 14.79	47.59 ± 19.16	1.586	0.01
Sex	Male	141 (46.5)	19 (48.7)	32 (45.1)	90 (46.9)	0.137	0.93
	Female	162 (53.5)	20 (51.3)	39 (54.9)	103 (53.4)		
Type of diabetes	T1DM	82 (27.1)	0 (0)	8 (11.3)	74 (38.3)	43.96	<0.001
	T2DM	217 (71.6)	39 (100)	61 (85.9)	117 (60.6)		
	LADA	1 (0.3)	0 (0)	0 (0)	1 (0.5)		
	MODY	3 (1.0)	0 (0)	2 (2.8)	1 (0.5)		
Duration of DM	>10 years	231 (76.2)	24 (61.5)	49 (69.0)	158 (81.9)	10.07	0.007
	<10 years	72 (23.8)	15 (38.5)	22 (31.0)	35 (18.1)		
Glycemic control	HbA1c >9%	79 (26.1)	0 (0)	6 (8.5)	73 (37.8)	62.09	<0.001
	HbA1c 7-9%	154 (50.8)	16 (41.0)	48 (67.6)	90 (46.6)		
	HbA1c <7	70 (23.1)	23 (59.0)	17 (23.9)	30 (15.5)		
Renal complications	eGFR <30 mL/min	9 (3.0)	0 (0)	0 (0)	9 (4.7)	9.72	0.04
	eGFR 30-45 mL/min	6 (2.0)	0 (0)	0 (0)	6 (3.1)		
	eGFR 45-60 mL/min	21 (6.9)	1 (2.6)	3 (4.2)	17 (8.8)		
	eGFR >60 mL/min	267 (88.1)	38 (97.4)	68 (95.8)	161 (83.4)		

Using IDF-DAR criteria, 39 patients (12.9%) were stratified into low-risk, 71 patients (23.4%) into moderate-risk, and 193 patients (63.7%) into high-risk categories. The high-risk group was characterized by younger age, higher HbA1c levels, greater prevalence of T1DM, and longer diabetes duration. Microvascular complications were absent in 237 (78.2%) of participants, while 64 (21.1%) had stable microvascular disease, 280 (92.4%) exhibited no signs of frailty or cognitive impairment, and 54 (17.8%) were physically active. Adherence to SMBG was 174 (57.4%) overall, highest in the high-risk group with 126 (65.3%) patients and lowest in the low-risk group with 18 (46.2%) patients.

Hyperglycemia, defined as a blood glucose level greater than 16.6 mmol/L, was reported in 141 participants (47%), which included 13 (33.3%) from the low-risk group, 26 (36.6%) from the moderate-risk group, and 103 (53.4%) from the high-risk group. Additionally, 10 individuals (3.4%) experienced DKA prior to Ramadan, with five (1.7%) occurring within the past three months and another five (1.7%) within the past year. During Ramadan, one patient with T1DM in the high-risk group was hospitalized for DKA. Medication adjustments were made for 120 (39.6%) patients before Ramadan, and 138 (45.5%) had adjustments during Ramadan. Significant differences in treatment modification were observed across risk categories (p < 0.001): 108 (78.3%) of high-risk, 23 (16.6%) of moderate-risk, and 7 (5.1%) of low-risk patients had modifications during fasting. Pre-Ramadan adjustments also differed significantly (p = 0.002): 90 (70%) high risk, 22 (18.3%) moderate risk, and eight (6.7%) low risk.

The reasons for breaking the fast during Ramadan are categorized by diabetes type (type 1 or type 2) and the number of fasting days attempted (one to 15 days, more than 15 days, all days, or none). Hypoglycemia was the most common reason for breaking the fast, particularly among 23 (71.9%) patients attempting to fast for more than 15 days. Patients with T1DM had a higher incidence of hypoglycemia (32; 39.0%) compared to T2DM (23; 10.6%).

A significant number of individuals broke the fast due to advice from their doctor, often for reasons other than hypoglycemia or hyperglycemia. This was more prevalent in T1DM (11; 13.4%) than in T2DM (two; 0.9%). Other reasons for breaking the fast included hyperglycemia, medication-related issues, gastrointestinal problems, and infections, though these were reported less frequently than hypoglycemia and physician recommendations, as shown in Table 2.

**Table 2:** Reasons for fasting fewer than 30 days reported in the post-Ramadan survey T1DM, type 1 diabetes mellitus; T2DM, type 2 diabetes mellitus

Group	Reason for breaking fasting	Total	Day fasted			
			1-15 days	More than 15 days	All days	Not a single day
T1DM	Hypoglycemia	32 (39.02)	8 (25.0)	23 (71.9)	0 (0)	1 (3.1)
	Physician advice	11 (12.8)	0 (0)	1 (9.1)	0 (0)	10 (90.1)
	Hyperglycemia	3 (3.5)	1 (33.3)	2 (66.7)	0 (0)	0 (0)
	Personal decision	1 (1.2)	0 (0)	0 (0)	0 (0)	1 (100)
T2DM	Hypoglycemia	23 (10.6)	0 (0)	21 (91.3)	0 (0)	2 (8.7)
	Gastrointestinal	1 (0.5)	1(100)	0 (0)	0 (0)	0 (0)
	Infective	2 (0.9)	0 (0)	2 (100)	0 (0)	0 (0)
	Other non-diabetes	3 (1.4)	2 (66.7)	1 (33.3)	0 (0)	0 (0)
	Hypertension	1 (0.5)	1(100)	0 (0)	0 (0)	0 (0)
	Physician advice	2 (0.9)	0 (0)	0 (0)	0 (0)	2 (100)
	Hyperglycemia	1 (0.5)	0 (0)	1 (100)	0 (0)	0 (0)
	Medication related	1 (0.5)	0 (0)	1 (100)	0 (0)	0 (0)
	Personal decision	1 (0.5)	0 (0)	0 (0)	0 (0)	1 (100)
	Psychiatric	1 (0.5)	1 (100)	0 (0)	0 (0)	0 (0)

**Figure 1:**
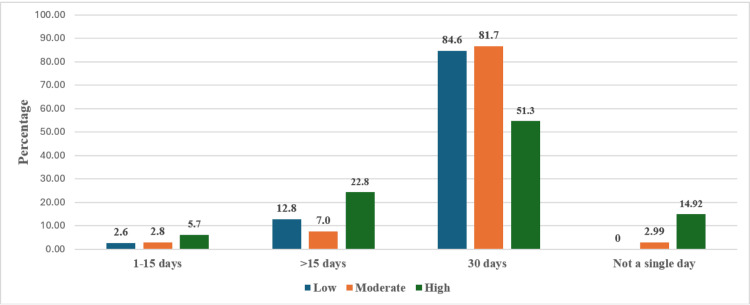
Percentage of days of fasting attained by individuals in the pre-Ramadan risk category

The percentage of fasting days attained by individuals in a pre-Ramadan risk category is illustrated in Figure 1, broken down by risk level (low, moderate, and high) and duration of fasting. A significant majority of individuals across all risk levels who attempted to fast did so for the full 30 days of Ramadan, with moderate-risk individuals showing the highest percentage (58; 81.7%), followed by low-risk (33; 84.6%) and high-risk (99; 51.3%). A smaller percentage fasted for more than 15 days but less than 30, with high-risk individuals showing the largest proportion (44; 24.31%). Very few individuals fasted for only one to 15 days, and a small percentage did not fast at all, with the highest proportion of non-fasters being in the high-risk group (27; 14.92%). This emphasizes that a notable percentage of individuals in the high-risk category refrained from fasting entirely, underscoring the influence of risk stratification on fasting behavior.

**Figure 2:**
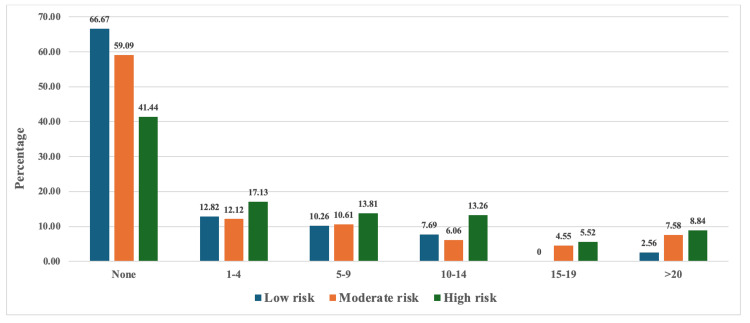
Percentage of patients experiencing any level of hypoglycemia throughout Ramadan, categorized by their estimated pre-Ramadan IDF-DAR risk score IDF-DAR, International Diabetes Federation-Diabetes and Ramadan

The majority of patients, regardless of risk level, experienced no hypoglycemic episodes during Ramadan. Specifically, the percentage of patients with no hypoglycemic events is highest in the low-risk group (26; 66.67%), followed by moderate (39; 59.09%) and then high-risk (75; 41.44%). Notably, among patients who experienced hypoglycemia, its prevalence exhibited a positive correlation with pre-Ramadan risk stratification scores. Furthermore, while absolute numbers were lower, the high-risk group demonstrated disproportionately higher proportions of patients with recurrent hypoglycemic episodes compared to low- and moderate-risk cohorts. This trend was especially notable in the categories of one to four episodes (31 patients, 17.13%) and more than 20 episodes (16 patients, 8.84%), as illustrated in Figure 2.

The ordinal regression model was statistically significant, indicating that the predictors reliably distinguished between the low, moderate, and high Ramadan risk categories. Significant predictors identified included age, duration of diabetes, hypoglycemia status, glycemic control (measured by HbA1C levels), and renal function (Table 3).

**Table 3:** Predictors of the low, moderate, and high risk Cox and Snell R² = 0.398; Nagelkerke R² = 0.478; McFadden R² = 0.285; Pearson chi-square test and deviance test p-value: 1.00 eGFR, estimated glomerular filtration rate

Predictor	Estimate (β)	p-value	95% CI (lower)	95% CI (upper)
Age	0.02	0.044	0.001	0.039
Duration of diabetes >10 years	Reference	-	-	-
Duration of diabetes ≤10 years	1.168	<0.001	0.529	1.808
Hypoglycemia (yes)	Reference	-	-	-
Hypoglycemia (no)	3.135	<0.001	1.967	4.303
HbA1C (<7%)	Reference	-	-	-
HbA1C >9%	-3.46	<0.001	-4.504	-2.416
HbA1C (7-9%)	-1.37	<0.001	-2.001	-0.739
Renal function = eGFR ≥60	Reference	-	-	-
Renal Function = eGFR <60	-2.602	<0.001	-3.81	-1.395

## Discussion

This study evaluated the IDF-DAR risk stratification tool’s efficacy in predicting adverse outcomes among Saudi patients fasting during Ramadan. A higher proportion of high-risk patients (63.7%) was observed compared to previous studies (10.1-56.9%) [12-16]. Variations in fasting practices may stem from differences in populations, settings, methodologies, and regional diabetes management. More individuals in moderate and high-risk categories fasted all 30 days, while some high-risk individuals did not fast at all. Low-risk individuals, primarily using non-insulin therapies, maintained HbA1c levels below 9%. Their favorable glycemic outcomes were attributed to a combination of factors, including a shorter duration of diabetes and the absence of significant disease progression. In contrast, high-risk patients, often with T1DM, longer disease duration, and insulin dependence, experienced recurrent hypoglycemia and HbA1c levels of over 9%. Notably, 51.3% of the high-risk group successfully fasted, consistent with prior studies [12-14], suggesting the IDF-DAR tool might overestimate fasting risks for high-risk patients, highlighting the need for personalized risk assessments.

In comparison with the study conducted at King Fahad Hospital in Al-Madinah Al-Munawarah [14], both studies validate the IDF-DAR risk stratification score and highlight the potential overestimation of risk in some high-risk patients. However, our study delves further into statistical predictive modeling of adverse outcomes, an aspect not thoroughly addressed in previous similar studies. This focus allows for a more nuanced understanding of the risk factors and patient profiles, contributing to improved individualized risk assessments and management strategies for diabetic patients fasting during Ramadan. Comparison with studies conducted across the globe reveals interesting contrasts and similarities. The overall incidence of hypoglycemia in our cohort was 18.2%, which is significantly higher than that reported in a study conducted in Bangladesh [13] but lower than the incidence observed in a study from Iran [17]. T1DM patients had more frequent hypoglycemia (39.0%) compared to those with T2DM (10.6%), aligning with previous findings [14]. Our study found that during Ramadan, 33% of T1DM patients and 25% of T2DM patients fasted for at least 15 days. These findings contrast with the EPIDIAR multi-country study (13 countries: Algeria, Bangladesh, Egypt, India, Indonesia, Jordan, Lebanon, Malaysia, Morocco, Pakistan, Saudi Arabia, Tunisia, and Turkey; n = 12,243), where 42.8% of individuals with T1DM and 78.7% of those with T2DM reported fasting for ≥15 days during Ramadan [18]. Hypoglycemia rates varied across risk categories: 48.7% in high-risk, 23.9% in moderate-risk, and 17.9% in low-risk groups (Figure 2). These rates differ from those reported in other studies [13,19], thereby highlighting the differences in risk of the various cohorts. For instance, a study conducted in Iran stratified diabetic patients based on the IDF-DAR guideline and found that 36.3% of the patients were categorized as low risk, 40% as moderate risk, and 23.7% of patients were categorized as high risk [17]. Hyperglycemia occurred in only 1.3% of our cohort, a notably low rate mirroring the DAR-BAN study [13]. When stratified by risk, prevalence was highest in the high-risk group (53.4%), compared to 36.6% (moderate-risk) and 33.3% (low-risk), aligning with Bangladeshi research demonstrating significantly elevated hyperglycemia risk in high- versus low-risk patients [13]. However, our overall rate contrasts with lower figures reported elsewhere, potentially reflecting differences in insulin dependence or adherence to fasting protocols [20,21].

Our study observed higher rates of medication adjustments pre-Ramadan (39.6%) and during Ramadan (45.5%)-exceeding previous reports, underscoring the importance of proactive pre-Ramadan care and patient education, particularly for high-risk populations [12,14]. SMBG practices also differed significantly by risk category: 10.3% of low-risk versus 72.4% of high-risk patients performed regular monitoring. The prediction model, which stratified fasting risk using age, diabetes duration, hypoglycemia history, HbA1c, and renal function, reinforces the need for individualized risk stratification and tailored management to mitigate complications. These findings align with frameworks like the Diabetes Canada (2018) position statement on Ramadan fasting and diabetes, which outlines pharmacotherapy and glucose-monitoring strategies to support safe fasting practices for Canadian Muslims with diabetes [22]. Future studies should expand risk models to incorporate comorbidities and fasting adherence patterns, enhancing their real-world clinical utility.

Strengths and limitations

This study is among the first to validate the IDF-DAR risk stratification tool in Saudi Arabia, providing valuable insights into managing diabetes during Ramadan. It effectively addresses a critical knowledge gap in a predominantly fasting population and includes a high-risk cohort, facilitating a comprehensive analysis of the tool’s performance in identifying patients at risk for complications. Furthermore, the study underscores the importance of patient education in enhancing health outcomes. Despite the useful insights provided by the IDF-DAR risk stratification tool, our study highlights its potential overestimation of risk in some high-risk patients. This could be due to cultural differences, patient education, and advanced diabetes management practices that allow some high-risk patients to fast safely. Additionally, the generalizability of the study is limited due to its population being sourced from tertiary care centers, which may not accurately reflect the wider diabetic population in Saudi Arabia. Additionally, reliance on self-reported data without caregiver monitoring can introduce biases. The use of different glucometers to monitor blood glucose at home across two centers may lead to variations in accuracy, which could affect the study’s reliability. Furthermore, the absence of post-Ramadan HbA1c data limits the evaluation of long-term glycemic control. Sparse data in some subcategories required the use of Fisher’s exact test, limiting the analysis’s power. Lastly, the lack of CGMS profiles among study participants could have provided more accurate data.

## Conclusions

This study highlights the complex relationship between religious practices and health management among people with diabetes during Ramadan. Emphasizes the need for comprehensive education. An individual treatment plan and continuous monitoring are needed to ensure safe fasting. The IDF-DAR risk stratification tool is effective in identifying diabetic patients at risk for complications during Ramadan fasting. However, some high-risk patients can fast safely. It highlights the limitations in the tool’s predictive accuracy. While the IDF-DAR tool is useful for risk categorization, individual assessments remain crucial. Future research should refine its predictive accuracy and address observed discrepancies.

To enhance the practical application of the IDF-DAR risk stratification tool, clinicians should consider supplementing the tool with thorough individual assessments to account for patient-specific factors such as comorbidities, duration of diabetes, and adherence to fasting guidelines. Awareness and education on safe fasting practices and potential risks associated with fasting for diabetic patients should be provided, alongside CGM and regular follow-ups, to ensure safe fasting and timely intervention if needed. Additionally, acknowledging cultural differences and tailoring the risk assessment and management strategies to fit the cultural context of the patient population is crucial for effective diabetes management during Ramadan.

## Figures and Tables

**Figure 3:**
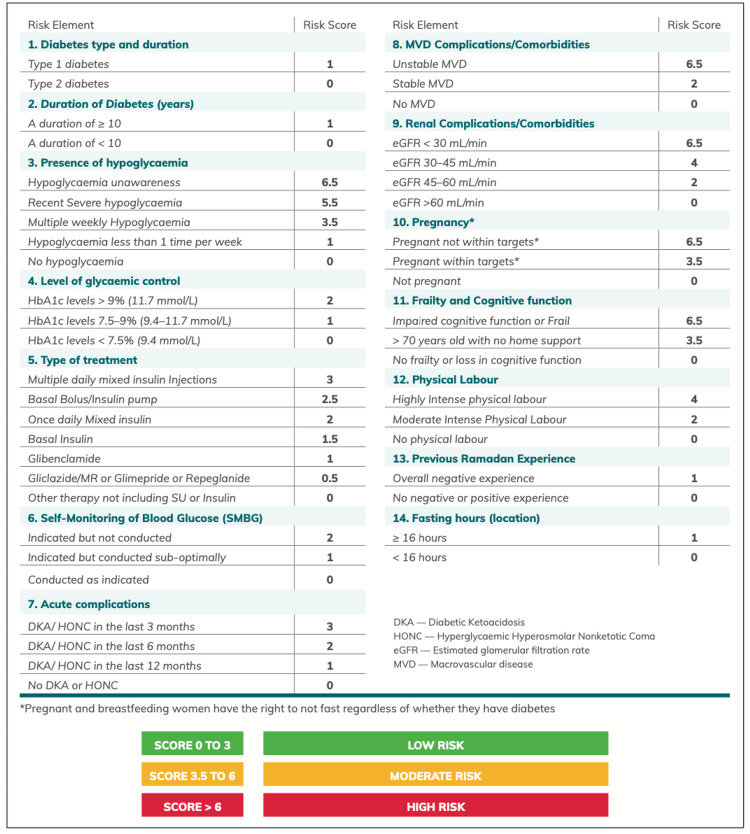
IDF-DAR assessment tool risk score IDF-DAR, International Diabetes Federation-Diabetes and Ramadan

## Appendices

Appendix A
